# Results of a Mass Media Campaign in South Africa to Promote a Sugary Drinks Tax

**DOI:** 10.3390/nu12061878

**Published:** 2020-06-23

**Authors:** Nandita Murukutla, Trish Cotter, Shuo Wang, Kerry Cullinan, Fathima Gaston, Alexey Kotov, Meena Maharjan, Sandra Mullin

**Affiliations:** 1 Vital Strategies, 100 Broadway, New York, NY 10005, USA; tcotter@vitalstrategies.org (T.C.); swang@vitalstrategies.org (S.W.); akotov@vitalstrategies.org (A.K.); mmaharjan@vitalstrategies.org (M.M.); smullin@vitalstrategies.org (S.M.); 2 Health-e News Service, Rosebank, Johannesburg 2196, South Africa; kerryjcullinan@gmail.com (K.C.); simjee.fathima@gmail.com (F.G.)

**Keywords:** obesity campaign, sugary drinks, mass media, tax, health harms, policy, communication, evaluations

## Abstract

Background: In South Africa, the increased consumption of sugary drinks has been associated with increased obesity rates. Mass media campaigns can play a crucial role in improving knowledge, shifting attitudes, and building support for government action on reducing sugary drink consumption. No study to date has evaluated the effectiveness of mass media campaigns on the health harms of sugary drinks in South Africa. Objective: The purpose of this study was to evaluate the impact of a mass media campaign on knowledge and attitudes around sugary drinks and on public support for a proposed tax on sugary drinks in South Africa. Methods: The “Are You Drinking Yourself Sick?” campaign aired in South Africa from October 2016 to June 2017 to shift attitudes toward sugary drinks, build personal risk perceptions of the health harms of consuming sugary drinks, and build public support for a proposed tax on sugary drinks. Campaign impact was measured in representative cross-sectional household surveys of adults ages 18 to 56. The surveys were conducted just prior to the launch of the campaign (*N* = 1000), from October 7 to 10, 2016, and immediately following its conclusion (*N* = 1000), from July 12 to 21, 2017. Campaign impact was assessed by comparing changes from the pre-campaign to the post-campaign period on key outcome indicators. In addition, the effect of campaign awareness was analyzed in logistic regression analysis of the post-campaign data. Results: The campaign was recalled by 55% of survey respondents, and 78% of campaign-aware respondents said that the campaign’s main message was “drinking sugary drinks can make you sick.” There were significant changes from the pre- to the post-campaign period in knowledge that sugary drink consumption can lead to obesity and related health problems and that sugary drinks contribute toward the obesity problem in South Africa. Campaign awareness was also significantly associated with increases in knowledge about the harms of sugary drinks, and in particular, on government action, including the proposed tax on sugary drinks. Discussion: Media campaigns are an effective intervention for obesity prevention. In addition to improving knowledge and shifting attitudes, media campaigns can effectively build public support for strong government action and therefore must be a component of a comprehensive obesity prevention approach.

## 1. Introduction

Obesity is a significant risk factor for a range of noncommunicable diseases, from cardiovascular diseases to diabetes and even some cancers [1,2,3]. The global obesity burden is growing, not just in high-income countries but also in low- and middle-income countries such as South Africa, [1] where almost 40% of women and 11% of men are obese [4].

Unhealthy diets have been found to be a chief contributor to rising obesity rates [5,6,7]. In particular, excess sugar consumption, often in the form of sugary drinks, is a major cause of obesity. It also increases the risk of diabetes, liver and kidney damage, heart disease, and some cancers [8]. Nevertheless, consumption of sugary drinks remains high globally [9], including in South Africa, where the rates of consumption have grown in both urban and rural areas [10,11,12,13]. 

Taxes on sugar-sweetened beverages (SSBs) that increase the price of the beverage and thereby reduce its accessibility, have emerged as an effective policy solution to counter increased consumption [14,15,16]. This has been demonstrated not only in modeling studies but also in real-world evaluations [17,18,19,20,21,22]. Even so, SSB taxes continue to face significant opposition from the food and beverage industry [23], potentially slowing their global adoption as an obesity prevention strategy [24,25]. Hence, as the South African government prepared to introduce a sugary drinks tax (also described as a Health Promotion Levy) in 2016, industry pressure against the tax was anticipated, and civil society organizations prepared to counter this pressure [26,27,28,29]. 

### Media Campaigns and Policy Support

A significant body of evidence has found mass media campaigns to play a critical role in advancing public health. Traditionally, media campaigns have been used to promote social and behavioral change, from the prevention and control of infectious diseases to family planning to, more recently, addressing noncommunicable diseases [30,31,32,33,34]. In the context of obesity prevention, media campaigns have successfully improved knowledge, personal relevance, or concern over obesity [35,36,37,38], and there is some evidence, although more limited, for the effectiveness of media campaigns in improving behavioral intentions and actions [38,39,40,41,42,43]. 

Beyond this, mass media campaigns have also played a crucial role in advancing public policies by changing attitudes and by channeling increased public concern over public health issues into support for government action to address those issues [30,44,45,46,47]. Indeed, in the context of obesity prevention, media narratives have had a significant impact on support for SSB taxes, and mass media campaigns have been effective in shifting attitudes toward favoring taxes on SSBs [42,48]. Thus, research suggests that mass media campaigns may serve as a crucial tool as governments seek to enact policies, particularly contested ones such as a sugary drinks tax. 

Based on this evidence, in 2016, and timed with a government proposal for a tax on sugary drinks, a mass media campaign on the health harms of sugary drinks consumption was implemented in South Africa. To our knowledge, no study to date has assessed the impact of a mass media campaign on knowledge, attitudes, and public support for government action on sugary drinks in South Africa. This study aims to address this gap in the literature by examining the impact of such a campaign on knowledge and attitudes around sugary drinks and public support for a proposed tax on sugary drinks in South Africa. We hypothesize that following the mass media campaign in South Africa, positive changes will be observed in knowledge and attitudes around the harms of sugary drink consumption as well as an increase in public support for the proposed tax to curb sugary drink consumption. 

## 2. Materials and Methods

### 2.1. South Africa’s “Are You Drinking Yourself Sick?” Campaign

In 2016, the Healthy Living Alliance (HEALA), a coalition of civil society organizations in South Africa committed to supporting policies to improve health and nutrition, with support from Vital Strategies, a global health organization, launched a campaign on the health harms of sugary drink consumption. Targeted at South African adults ages 18 to 45, this campaign sought to build the public agenda for obesity prevention by (a) shifting attitudes toward sugary drinks, (b) building personal risk perceptions of the health harms of consuming sugary drinks, and (c) building public support for strong government action, in particular a tax on sugary drinks.

With support from Vital Strategies, HEALA pretested public service announcement (PSA) concepts with the target audience, including several PSAs from the United States (U.S.) and Mexico, to ensure their adaptability, cultural appropriateness, and potential effectiveness. In particular, noting the potentially stigmatizing nature of messages on obesity prevention [49], special attention was paid to ensuring that the PSAs did not stigmatize overweight or obese individuals. After pretesting, “Are You Drinking Yourself Sick?” was selected as the campaign’s core message and title. The campaign consisted of two 30-s PSAs that use family- and child-focused messaging. The first PSA, launched in October 2016, features a father and daughter drinking a sugary drink. As they swallow, we see via animation how sugar is dumped into the bloodstream with every sip and leads to fat buildup in and around vital organs bringing on obesity, heart disease, and type 2 diabetes. The second ad, “Journey,” launched in May 2017, follows a woman throughout her day as she drinks several sugary drinks. The PSA reinforces that the amount of sugar she consumed over one day—50 teaspoons—increases the risk of diseases that can take people away from their families and loved ones. 

**Figure 1:**
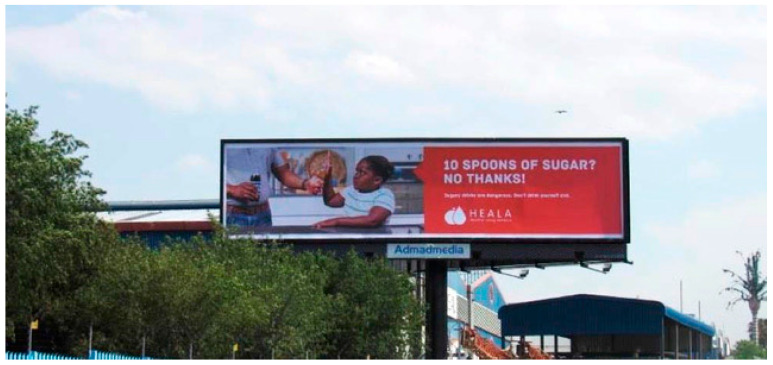
Photograph of outdoor media (billboard) from the campaign, “10 spoons of sugar? No thanks!”

**Figure 2:**
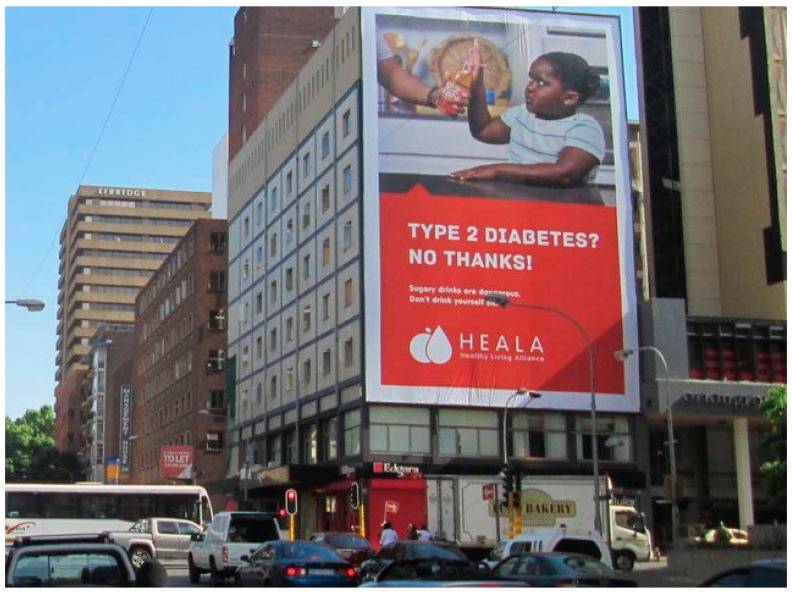
Photograph of outdoor media (billboard) from the campaign, “Type 2 diabetes? No thanks!”

**Figure 3:**
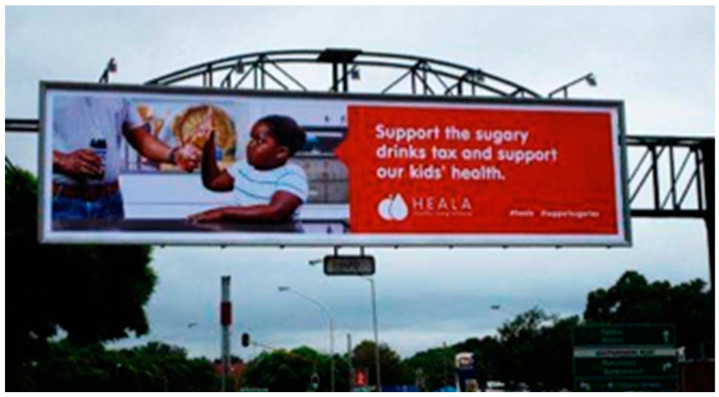
Photograph of outdoor media (billboard) from the campaign, “Support the sugary drinks tax and support our kids’ health!”

From October 2016 to June 2017 (with the exception of late December to early January), the campaign PSAs ran on multiple mass media outlets, including television and radio; outdoor (billboards, posters) and print (newspaper) media advertisements followed. The television and radio PSAs were broadcast in English and indigenous languages, isiZulu and isiXhosa, on major television and radio channels. Outdoor advertisements consisted of billboards and posters with an image of a child holding her hand up to stop someone from giving her a sugary drink that included the key messages “You wouldn’t give your child 10 spoons of sugar, would you?” and “Type 2 diabetes? No thanks” and provided additional information on the health risks of sugary drinks, such as “Sugary drinks are dangerous” and “Sugary drinks lead to fat build-up in and around vital organs, bringing on obesity, type 2 diabetes, and heart disease” (Figure 1 and Figure 2). In addition, another set of outdoor media paired the image of the child with tax-focused messaging that called for support for the sugary drinks tax, including “Support the sugary drinks tax and support our kid’s health” (Figure 3). The outdoor advertisements were placed mainly in urban areas in three provinces and key political locations, including the path between the national Parliament in Cape Town and the capital, Pretoria. Newspaper advertisements included key messages “Whose side are you on?” and “10 teaspoons of sugar,” encouraging policymakers to support the tax in order to support children’s health, address industry opposition to the tax, and urgently address rising diabetes rates.

The media campaign was a significant part of a broader effort, which also included evidence-based research, a strategic advocacy campaign, and stakeholder and community engagement, to build political and public support for a tax on sugary beverages in South Africa [50]. The evidence-based research was conducted by a research-to-policy unit at the University of the Witwatersrand, named PRICELESS (or “Priority Cost Effective Lessons for System Strengthening South Africa”). Backed by this research, PRICELESS, the Public Health Association of South Africa (PHASA), HEALA, and other advocates of the tax invested in a strategic advocacy campaign, which included stakeholder and community engagement, to garner support for a sugary drinks tax from government officials, civil society and academics. An open letter in support of the sugary drinks tax was published in the Sunday Times, and multiple public hearings with presentations in support of taxing sugary beverages were held. As with the mass media campaign, these efforts sought to encourage national conversations about the health harms of sugary beverages and build political and public support for the sugary drinks tax to address obesity.

A campaign evaluation was subsequently undertaken to measure the campaign’s effectiveness in increasing knowledge, shifting attitudes, and building support for the SSB tax. Based on the literature, behavioral changes were not anticipated from a campaign of relatively short duration, but they were nonetheless assessed. Campaign impact was measured in representative cross-sectional face-to-face household surveys of adults ages 18 to 56 to measure campaign-related changes in knowledge, attitudes, and behaviors toward sugary drinks and support for government action, particularly a sugary drinks tax to curb the rising obesity epidemic in the country. The surveys were conducted by Genesis Analytics just before the launch of the campaign (the “pre-campaign” survey) from October 7 to 10, 2016, and immediately following its conclusion (the “post-campaign” survey) from July 12 to 21, 2017.

### 2.2. Sample and Data Collection

The pre- and post-campaign surveys were conducted face-to-face in cities in three provinces: Gauteng, Kwa-Zulu Natal, and Western Cape. These provinces were chosen purposively in areas where the campaign was confirmed to have aired according to the media planners’ reports. Within the provinces, a multistage probability sampling procedure was implemented. Metro areas and cities within the survey provinces were first chosen. Households in each survey site were then selected from the Nielsen GeoFrame, a database of 6 million addresses arranged alphabetically by suburb and within suburb by street name. At each household, a screener questionnaire was implemented, and a Politz grid was used to select one respondent from multiple eligible respondents. For unavailable households, three callbacks on different days were made per household; thereafter, substitution was allowed from households in the immediate vicinity. Respondent eligibility criteria included ages between 18 and 56 years and resident in that location for over six months. The final samples in both the pre- and post-campaign surveys included 1,000 respondents.

### 2.3. Questionnaire and Measures

The questionnaire was administered face-to-face in English, Afrikaans, Zulu, Sotho, Xhosa, and Tswana via computer-assisted personal interviewing (CAPI). The following were the key measures in the questionnaire (the order of presentation in the questionnaire differed from what is presented here in order to minimize order effects).

Recall of messages on the harms of sugary drinks was measured in two ways: first, respondents were asked to recall any messages they had seen in the prior three months about the health harms of sugary drinks. They were also asked to indicate where they had come across those messages and what main messages they recalled. Later in the interview, campaign ad recognition was measured by showing participants images from the “Are You Drinking Yourself Sick?” campaign, including images from the TV PSA.

Reactions to the campaign were then assessed by asking campaign-aware respondents how strongly they agreed or disagreed with a series of statements about the campaign, including that it was (1) believable; (2) relevant; (3) taught something new; (4) created concern about the harms of sugary drinks; (5) increased interpersonal communication about the harms of sugary drinks; and (6) increased support for government action. Each of these statements was evaluated separately and strength in agreement or disagreement for each statement was measured using a five-point Likert scale.

Knowledge about obesity, and sugary drinks as a risk factor for obesity, was assessed through a series of yes or no questions that probed respondents’ knowledge of the main contributors of obesity and of the illnesses that might be caused by obesity or sugary drinks. Attitudes toward obesity and sugary drinks were assessed by measuring respondents’ agreement (on five-point Likert scales) with a series of statements on obesity and sugary drinks. Specifically, the questions assessed respondents’ attitudes on: the extent to which they considered obesity/sugary drinks to be a serious public health problem in South Africa; their attitudes toward sugary drinks as a cause of obesity; and the role of advertising and food labels on levels of sugary drinks consumption among adults and children.

A series of questions was asked to assess support toward government action to reduce obesity, including a sugary drinks tax. Respondents were asked how strongly they agreed or disagreed with a series of statements about government action to reduce obesity. Strength in agreement or disagreement for each statement was measured using a five-point Likert scale. Support for a sugary drinks tax itself was assessed in two ways: first, participants were asked how strongly they support or oppose a tax on sugary drinks if the money collected was to be invested in public programs. Later, they were specifically asked about the South African proposal as follows: “As part of its plan to address obesity in South Africa, the Department of Health recommends increasing the tax on sugary drinks, and the Department of Finance/Treasury has proposed a tax on sugar content that amounts to a roughly 20% tax on sugary drinks. Do you support or oppose the government’s proposal to tax sugary drinks?”

Behaviors pertaining to sugary drinks and intentions regarding their consumption in the future were measured.

Sociodemographic information was also measured, including gender, age, socioeconomic status (low, medium, and high), and parent/primary caregiver status (are you a parent or primary caregiver to children under the age of 16 who reside with you?) in the screener questionnaire. In the main survey, additional sociodemographic information was collected, including frequency of vigorous physical activity (vigorous physical activity three days or more a week—defined as activities that make people breathe much harder than normal and may include heavy lifting, digging, aerobics, or fast bicycling and only those that they did for at least 10 min at a time), fruit and vegetable intake in the last seven days (consumed fruits and vegetables over three times a week), and frequency of television watching (watched more than four hours weekly).

### 2.4. Data Analysis

Data were weighted to adjust for oversampling or any mismatch between the sample profile and the estimated universe. The source of weighting was the All Media and Products Study (AMPS) (Jan 2015–Dec 2015). The data were then analyzed using IBM SPSS Version 25.

Two sets of comparisons were made to assess campaign impact. First, pre-campaign data were compared with post-campaign data to detect changes over time. These comparisons between proportions were made using chi-square tests. For continuous variables, a t-test was used for the comparison. Second, in order to identify campaign-attributable impacts, the respondents were categorized according to whether they were “campaign aware” or “campaign unaware.” All respondents who recalled either of the ads from TV, which was the predominant campaign channel, were categorized into the “campaign aware” group, while all others were categorized as campaign unaware. Hence, those that recalled the campaign only via one of the complimentary newspaper advertisements were categorized as “campaign unaware.” The comparisons between the “campaign aware” group and the “campaign unaware” group were first made using chi-square test for categorical variable or using a t-test for continuous variables. Then, campaign awareness was regressed onto dichotomized measures of knowledge, attitudes, government policy support, and behavioral items. Covariates for the logistic regression analysis included age, gender, socioeconomic status, and frequency of watching television. The significance level for all tests was set to *p* < 0.05.

## 3. Results

### 3.1. Sample Characteristics

Table 1 presents the demographic characteristics of the different pre- and post-campaign samples. The pre- and post-campaign survey participants were similar in terms of age, gender, education, and socioeconomic status. However, as indicated in Table 1, significant differences were observed between the pre- and post-campaign survey participants. Respondents interviewed in the post-campaign period were significantly more likely than those in the pre-campaign period to report being unemployed (37% vs. 44%); consume lower levels of fruits and vegetables (58% in the pre- vs. 53% in the post-campaign period consumed fruits and vegetables over three times a week); to report higher levels of TV watching (25% vs. 42% watched more than four hours weekly); to report higher levels of physical activity (40% vs. 57% engaged in vigorous physical activity more than three times in a week); and to report poorer health status (15% pre-campaign vs. 24% post-campaign).

### 3.2. Recall of Messages About Harms of Sugary Drinks

There was a statistically significant increase from pre- to post-campaign in the recall of messages about health harms of sugary drinks (13% vs. 29%, *p* < 0.001) and in the frequency with which these messages were encountered (32% vs. 50%, “often” came across these messages, *p* = 0.001). A total of 55% of participants recalled at least one of the campaign ads through any of the media used: 46% of respondents recalled the first TV ad; 32% recalled the second TV ad; 15% recalled the newspaper ad “Whose side are you on?;” 22% recalled the newspaper ad “10 teaspoons of sugar.” Of those who recalled the campaign, 78% said that the campaign’s main message was that “drinking sugary drinks can make you sick.”

There were sociodemographic differences in campaign recall. As indicated in Table 2, campaign recall varied by age, gender, employment status, socioeconomic status, television watching, and self-reported body mass index (BMI). Specifically, campaign recall was higher among women than among men; and among younger rather than older adults; those who described themselves as students or “other” compared to other employment categories; those with a high socioeconomic status compared to those with a medium or low socioeconomic status; those that watched more than four hours of TV a week; and those who reported their BMI as normal compared to either under- or over-weight adults.

### 3.3. Reactions to the Campaign

Among post-campaign survey participants, those who were aware of the campaign rated it positively (Table 3). The vast majority of participants who recalled the campaign agreed the campaign was believable, relevant to them, taught them something new, and made them stop and think. In addition, among those aware of the campaign, 85% agreed the campaign made them feel concerned about the impact of sugary drinks on their health and motivated them to reduce their consumption of sugary drinks. Among parents/caregivers who recalled the campaign, about eight out of ten participants agreed that the campaign made them motivated to reduce their child’s consumption of sugary drinks (82%). Over two-thirds of participants who were aware of the campaign reported they were likely to reduce sugary drink consumption as a result of seeing the campaign (68%).

The campaign also generated interpersonal communication about the health harms of sugary drinks: 90% of campaign-aware respondents said that they “would like others to see this ad;” 87% said, “it provides a public service/it is in public’s interest to watch it.” Nearly three-quarters of the campaign-aware respondents (74%) said that they “discussed the campaign with someone else.” Of those, 33% did so with their family, 24% with friends, 11% with colleagues, and 6% discussed it with a doctor or a health worker.

Finally, 81% of campaign-aware respondents said that the campaign made them more supportive of government actions to reduce sugary drinks consumption, and 83% supported ads like this one on the health effects of sugary drinks being shown on TV (Table 3).

### 3.4. Knowledge and Attitudes

#### 3.4.1. Changes from the Pre- to the Post-Campaign Period

In both pre- and post-campaign periods, a similarly high percentage of respondents said that overweight/obesity among adults in South Africa was “very much” a problem (73% vs. 75%). However, there was a significant increase from the pre-campaign to the post-campaign period in the percentage of respondents who said that the following were very much a problem in South Africa: overweight/obesity among children (61% vs. 67%), undernutrition (55% vs. 61%), and oral health (49% vs. 56%) (Table 4).

Knowledge that consuming sugary drinks can lead to being overweight or obese increased from the pre-campaign to the post-campaign period (66% vs. 71%), and there was a significant increase in the percentage of South African adults who listed junk food (92% vs. 97%) and sugary drinks (76% vs. 90%) as among the top three contributors to obesity in South Africa. Compared to the pre-campaign period, there was increased agreement that too much sugar can cause severe health problems (87% vs. 90%) and that sugary drinks or junk food advertisements encourage unhealthy diets among children (76% vs. 82%). However, a large and significantly increased percentage of adults continued to believe that as long as they exercised, too much sugar would not harm their health (56% vs. 64%).

#### 3.4.2. Impact of Campaign Awareness Within the Post-Campaign Period

As described in Table 4, within the post-campaign period, campaign awareness was associated with significantly increased knowledge that overweight/obesity increases the risk of serious illness in adults (86% vs. 89%) and children (84% vs. 89%). Campaign awareness was likewise associated with knowledge that the consumption of sugary drinks leads to being overweight or obese (66% vs. 75%) and that it increases the risk of suffering from obesity (62% vs. 68%) and dental problems (48% vs. 60%). Finally, campaign-aware respondents were less likely to agree with the inaccurate statement, “as long as one exercises, too much sugar will not be harmful” (68% unaware vs. 61% aware), and to report they are concerned about the effects of sugary drinks on health (53% vs. 62%).

### 3.5. Support for Government Action

#### 3.5.1. Changes from the Pre- to the Post-Campaign Period

Support for government action to solve the problem of obesity in South Africa increased from the pre- to the post-campaign period (73% vs. 84%). As indicated in Table 5, support grew for government efforts to increase children’s access to healthy foods and drinks (80% vs. 84%); for the passage and enforcement of policies that discourage junk food and sugary drinks (67% vs. 76%); for restrictions on the sale and/or provision of sugary drinks and junk food in schools (66% vs. 74%); and for bans/restrictions on advertising and/or marketing of sugary drinks and junk foods targeted at children (63% vs. 69%), including on school property and at school activities (61% vs. 69%). There were statistically significant increases in support for a higher tax on sugary drinks, particularly if the money collected was invested in public programs (62% pre-campaign vs. 70% post-campaign). On the specific sugary drinks tax proposal by the South African Department of Health, support for it increased significantly from the pre-campaign to the post-campaign period (43% pre-campaign vs. 58% post-campaign).

#### 3.5.2. Impact of Campaign Awareness within the Post-Campaign Period

Campaign awareness was associated with significantly increased support for government action to address obesity in South Africa (see Table 5). Campaign-aware respondents were significantly more likely than campaign-unaware respondents to support communication campaigns that warn about the damage of sugary drinks and junk food on health (78% vs. 83%); restrictions on the sale and/or provision of sugary drinks and junk food in schools (70% vs. 77%); bans or restrictions on advertising and/or marketing of sugary drinks and junk foods targeted at children (65% vs. 72%); and bans on marketing or advertising of junk food/sugary drinks on school property and at school activities (65% vs. 73%). Campaign-aware respondents were significantly more likely than unaware respondents to support a tax on sugary drinks if the money collected was invested in public programs (65% vs. 74%). Finally, on the specific sugary drinks tax proposal by the South African Department of Health, campaign-aware respondents were significantly more likely to support it than campaign-unaware respondents (54% vs. 62%).

### 3.6. Behavioral Intentions and Behaviors

#### 3.6.1. Changes from the Pre- to the Post-Campaign Period

As presented in Table 6, there was an increase in the percentage of South Africans who thought often of the harms to their health from consuming sugary drinks (44% vs. 50%) and intended to reduce their consumption of sugary drinks (66% vs. 74%). A significantly greater percentage also expressed an interest in getting their family to reduce their consumption of sugary drinks (67% vs. 79%) and intended to reduce how often they offered sugary drinks to a child (65% vs. 74%). There was increased self-reported confidence in their own ability to reduce their consumption of sugary drinks (65% vs. 73%) and an increase in expressed urgency to do so within the next week (52% in the pre-campaign vs. 58% in the post-campaign period). There was a significant increase from the pre-campaign to the post-campaign period in the percentage who said that they had reduced their consumption of sugary drinks compared to six months before (23% vs. 27%).

#### 3.6.2. Impact of Campaign Awareness Within the Post-Campaign Period

Within the post-campaign period, campaign awareness was only associated with an increase in the consumption of water compared to six months before (39% vs. 45%). There were no other associations between campaign awareness and behaviors noted.

## 4. Discussion

The study findings demonstrated that the South African “Are You Drinking Yourself Sick?” campaign performed as intended. More than half the population surveyed recalled the campaign, and it was well received. Campaign-aware respondents accurately recalled the campaign’s message, they found it to be believable and relevant, and it increased their concern about the harms of sugary drinks. Over half of campaign-aware respondents discussed its key message with others, and 81% of campaign-aware respondents said that the campaign made them more supportive of government action to reduce sugary drink consumption.

There were significant improvements in knowledge, attitudes, and behaviors between the pre- and post-campaign periods. There was a significant increase in recognition of the problem of childhood obesity, increased knowledge of the harms of sugary drinks on the health of adults and children, and increased knowledge that sugary drinks contribute to the obesity problem in South Africa.

While the changes from the pre- to the post-campaign period may arguably have been the result of concurrent activities beyond the campaign, the comparison of campaign-aware and campaign-unaware respondents in the post-campaign data suggests the independent impact of the campaign on these outcomes. Even after potential confounders were controlled for, the data showed that campaign-aware respondents were significantly more likely than unaware respondents to demonstrate increased knowledge about the serious health risks of overweight/obesity and to express increased knowledge and concern about the harms of sugary drinks. In this regard, campaign awareness also played an important role in reversing the mistaken belief that exercise can protect against the harm of sugary drinks. Campaign awareness in the post-campaign period was associated with a decreased tendency for people to believe that exercise would have this protective effect.

Most importantly, this study found significant increases in support for government action, the primary objective of the campaign. These increases in support were observed from the pre- to the post-campaign period: for instance, there was a significant increase in South Africans’ support for government actions that discourage the consumption of sugary drinks and junk foods and in support for a tax on sugary drinks if the money collected was invested in public programs.

Participants were also asked more specifically about the proposal by the Department of Finance. While the expression of support to this more pointed question may be expected to be lower since conceivably many participants would not believe themselves to be fully informed of the proposal, the majority expressed support for this proposal and the proportion who expressed support grew significantly from the pre- to the post-campaign period. Furthermore, campaign awareness was found to have an independent association with support for government action. Within the post-campaign period, even after controlling for potential confounders, campaign-aware respondents were significantly more likely than unaware respondents to express increased support for strong government action, and for the sugary drinks tax, including the Department of Finance proposal.

Finally, while there were significant improvements in behavior between the pre- and the post-campaign periods, there was no independent association between campaign awareness and behavioral changes in the post-campaign data alone, suggesting that the behavioral changes may have been the result of the confluence of other activities surrounding the sugary drinks tax proposal and the increased interpersonal communication that may have been generated by the campaign and these other activities. In fact, increased interpersonal communication has been established as an important outcome and co-benefit of media campaigns.

That said, campaign awareness was independently associated with one item—increased water consumption—which could potentially be explained by the presence of an unrelated but extensive social media campaign promoting water at around the same time. These findings are consistent with the agenda-setting objective of the campaign, and they suggest the important benefits that might accrue from the increased interpersonal communication and narrative shifts that occur during media campaigns.

This study extends the literature on the important role of media campaigns in addressing behavioral risk factors [30,31,32,33,34]. It replicates findings from high-income countries, and as in other public health applications, it shows that media campaigns for obesity prevention can play a crucial role in improving awareness, and changing knowledge, attitudes, and social conversations around obesity and its behavioral risk factors [30,31,32,33,34]. Additionally, this study extends the literature by suggesting the important agenda-setting function that can be served by media campaigns for obesity prevention. Fiscal policies, such as the sugary drinks tax, have typically met with significant opposition and expensive public relations campaigns from the food and beverage industry that have sought to turn public opinion against these public health fiscal measures [23,24,25]. This study demonstrates that public health practitioners can successfully use evidence-based media campaigns that present the public health case—in this case, the harms of sugary drinks and the need to reduce its consumption in South Africa—to generate social conversations, build public engagement, and thereby generate support for such policies. Indeed, as described above, the independent association between campaign awareness and increased support for government action highlights the important need for policy proposals, such as sugary drinks taxes, to be paired with complementary, evidence-based media campaigns that highlight the public health case for such measures [30,44,45,46,47].

There were a few limitations to this study that are important to consider. First, despite the application of similar methods, the study sample in the post-campaign period varied in demographic profile compared to the sample in the pre-campaign survey. These variations were to an extent controlled for in the regression analysis that examined the association between campaign awareness and study outcomes. More importantly, the nature of the variation between the samples would have made the campaign impact harder—not easier—to detect. In fact, a common critique of media campaigns has been that their impact tends to be greater among higher-educated groups with greater socioeconomic status. Thus, this study’s evidence of improvement, and the association between campaign awareness and study outcomes, is particularly noteworthy. Second, the definition of “campaign awareness” in this study was restricted to those who recalled the TV ad alone. This was done to reflect the main channel of the communication campaign, but conceivably respondents who recalled the newspaper ads—and those who were exposed to the campaign but did not recall it in response to the survey question—may have been included in the campaign-unaware group, rendering this group a mix of those who were truly unaware of the campaign and those with some recall of the campaign. That said, it is a frequent limitation of campaign evaluations that rely on survey recall questions that those with campaign exposure but no recall cannot be distinguished from those with no exposure to the campaign. From the perspective of the assessment of campaign impact, however, this limitation would make it harder to detect campaign impact. Hence, the inherent bias is toward weakening the detection of campaign impact and not its converse, making the observation of campaign impacts particularly noteworthy. Finally, this campaign evaluation, as others, may be prone to the “selective attention” bias—the tendency of greater recall among those sympathetic toward or prone toward the campaign’s cause. However, the consistency in findings between the pre- and post-campaign comparisons and the regression analysis on most measures, and the fact that the post-campaign sample had more unhealthy dietary habits and poorer self-reported health status, and were thus less—not more—inclined toward the campaign, lends support for the effectiveness of the campaign.

## 5. Conclusions

In conclusion, this study supports the critical role that media campaigns can play to engage citizens and build public support for government policy actions to reduce obesity, in particular through sugary drinks taxes. Media campaigns can improve knowledge, attitudes, and generate public conversations. By communicating public health needs, such as the need to reduce sugary drinks consumption to reduce obesity, they can help governments build public support for action. Consistent with the literature on the use of media campaigns to promote healthy behaviors [30], this study suggests the need for media campaigns in a comprehensive obesity strategy.

## Figures and Tables

**Table 1:** Demographic characteristics of final study sample in the pre- and post-campaign periods.

	Pre-Campaign	Post- Campaign	*p*-Value
	(*n* = 1000)	(*n* = 1000)	
Age (Mean ± SD) (years)	34 ± 10.49	35 ± 10.65	0.041 *
Women (%)	50	50	1.000
Parent/primary care giver to children under 16 (%)	59	55	0.058
Education (%)			
High school and below	83	86	0.100
Above high school	17	15	
Employment status (%)			
Unemployed	37	44	0.019 *
Employed	55	49	
Student	5	4	
Other	4	3	
Socioeconomic status (%)			
Low	9	12	0.252
Medium	66	64	
High	25	25	
Fruit and vegetable intake in the last seven days (%)			
Two or fewer times	42	47	0.048 *
Three or more times	58	53	
Frequency of watching television in the last seven days (%)			
Less than four hours	75	58	<0.001 *
More than four hours	25	42	
Vigorous physical activity in the last seven days (%)			
Two or fewer days	60	43	<0.001 *
Three or more days	40	57	
Body mass index (%)			
Underweight	1	3	0.122
Normal weight	30	33	
Overweight	25	20	
Obese	44	44	
Self-rated health status (%)			
Poor/fair health	15	24	<0.001 *
Good/excellent health	85	76	

* Significant difference at *p* < 0.05.

**Table 2:** Demographic characteristics of campaign-aware and campaign-unaware respondents in the post-campaign period.

	Post-Campaign		
	Unaware (*n* = 453)	Aware (*n* = 547)	*p*-Value
Age (Mean ± SD) (years)	36 ± 10.50	35 ± 10.77	0.010 *
Gender (%)			
Men	52	49	<0.001 *
Women	39	61	
Parent/primary caregiver of children under 16 (%)			
Yes	44	56	0.287
No	47	53	
Education (%)			
High school and below	45	55	0.344
Above high school	49	51	
Employment status (%)			
Unemployed	45	55	0.078
Employed	47	53	
Student	33	67	
Other	58	43	
Tobacco user (%)			
Yes	45	55	0.982
No	45	55	
Socioeconomic status (%)			
Low	52	48	0.023 *
Medium	47	53	
High	39	62	
Vigorous physical activity in the last seven days (%)			
Two or fewer days	50	50	0.102
Three or more days	44	56	
Fruit and vegetable intake in the last seven days (%)			
Two or fewer times	42	58	0.056
Three or more times	48	52	
Frequency of watching television in the last seven days (%)			
Less than four hours	51	49	<0.001 *
More than four hours	38	62	
Body mass index (%)			
Underweight	46	54	0.212
Normal weight	37	63	
Overweight	47	53	
Obese	48	52	
Self-rated health (%)			
Poor/fair health	48	52	0.425
Good/excellent health	45	52	

* Significant difference at *p* < 0.05.

**Table 3:** Reactions to the campaign among those who recalled it through any medium.

Reactions to the Campaign	Campaign Aware (*n* = 547)
% that agreed that the campaign…	
Was believable	86
Was relevant to me	79
Taught me something new	83
Made me stop and think	82
Made me feel concerned about the impact of sugary drinks on my health	85
Made me motivated to reduce my consumption of sugary drinks	85
Made me motivated to reduce my child’s consumption of sugary drinks	82
Made me more supportive of government action to reduce sugary drink consumption in my country	81
I would like others to see this ad	90
This ad provides a public service/it is in the public’s interest to watch it	87
% that discussed the campaign with…	
Family	33
Friends	24
Colleagues	11
Doctor or health worker	6
Likelihood of reducing the amount of sugary drinks consumed as a result of seeing the campaign (% likely)	68
Support for ads like this one on the health effects of sugary drinks being shown on TV (% support)	83

**Table 4:** Knowledge and attitudes toward obesity and sugary drinks among respondents in the pre-campaign and post-campaign periods, including a comparison of post-campaign respondents who were aware and unaware of the campaign.

	Pre-Campaign	Post-Campaign		Post-Campaign				
			*p*-Value	Unaware	Aware	*p*-Value	Adj. OR^ (95% CI)	*p*-Value
	(*n* = 1000)	(*n* = 1000)		(*n* = 453)	(*n* = 547)			
In your opinion, how much of a problem, if at all, are the following in South Africa? (% Very much/A lot)								
Overweight or obesity among adults	73	75	0.169	73	77	0.155	1.25 (0.93, 1.68)	0.135
Overweight or obesity among children	61	67	0.003 *	65	69	0.162	1.22 (0.93, 1.60)	0.162
Under-nutrition	55	61	0.004 *	61	61	0.977	1.02 (0.78, 1.33)	0.888
Oral health	49	56	0.001 *	58	55	0.308	0.88 (0.68, 1.14)	0.324
Does being overweight or obese increase risk of…? (% Somewhat/greatly)								
An adult’s developing serious illnesses	87	88	0.774	86	89	0.105	1.56 (1.05, 2.30)	0.028 *
A child’s developing serious illnesses	78	87	<0.001 *	84	89	0.021 *	1.49 (1.02, 2.18)	0.040 *
Diabetes	96	94	0.008 *	92	95	0.153	1.50 (0.89, 2.54)	0.131
Hypertension	96	94	0.050 *	94	93	0.255	0.76 (0.45, 1.29)	0.313
Heart disease, including heart attacks	94	93	0.647	92	95	0.069	1.61 (0.96, 2.70)	0.069
Cancer	64	65	0.608	65	65	0.790	0.97 (0.74, 1.26)	0.803
Premature death	89	86	0.121	86	87	0.853	1.11 (0.76, 1.60)	0.600
In your opinion, to what extent does the consumption of sugary drinks lead to being overweight or obese? (% Very much/a great deal)	66	71	0.007 *	66	75	0.002 *	1.62 (1.22, 2.16)	0.001 *
Does drinking sugary drinks increase the risk of suffering from…? (% Somewhat/greatly)								
Diabetes	71	71	0.126	69	73	0.084	1.27 (0.96, 1.69)	0.094
High blood pressure	65	70	0.017 *	67	71	0.175	1.28 (0.97, 1.69)	0.088
Cancer	41	47	0.010 *	45	48	0.437	1.15 (0.89, 1.49)	0.288
Obesity	59	66	0.003 *	62	68	0.055	1.29 (0.99, 1.69)	0.061
Dental problems	45	55	<0.001 *	48	60	<0.001 *	1.64 (1.27, 2.13)	<0.001 *
Top three contributors to obesity are: (% that mentioned)								
Eating junk foods	92	97	<0.001 *	97	97	0.736	0.98 (0.48, 2.00)	0.952
Drinking sugary drinks	76	90	<0.001 *	89	91	0.426	1.04 (0.68, 1.58)	0.860
Lack of exercise	80	88	<0.001 *	86	89	0.088	1.41 (0.96, 2.07)	0.080
Agreement with statements about obesity/sugary drinks (% agree)								
Childhood obesity is a problem in my country	74	80	0.001 *	82	78	0.235	0.89 (0.64, 1.23)	0.464
Too much sugar can cause severe health problems	87	90	0.020 *	91	90	0.353	0.82 (0.53, 1.27)	0.368
Advertising of sugary drinks and junk foods encourages children toward unhealthy diets	76	82	0.001 *	81	83	0.517	1.13 (0.81, 1.58)	0.462
As long as I exercise regularly, too much sugar will not harm my health	56	64	<0.001 *	68	61	0.017 *	0.72 (0.55, 0.95)	0.019 *
The nutrition labels on food and drinks help me to make healthy choices	77	79	0.254	80	79	0.543	0.87 (0.64, 1.20)	0.394
Concerned about the effect of drinking sugary drinks on your health? (% concerned)	--	58	--	53	62	0.007 *	1.52 (1.17, 1.97)	0.002 *

* Significant difference at *p* < 0.05. Abbreviations: OR, Odds Ratio; CI, Confidence Interval. ^ Covariates adjusted for include age, gender, living standard measure, frequency of watching TV. A significant adjusted OR indicates that even after confounding factors have been taken into account, the odds of the “aware” group’s reported intentions/behaviors are significantly different from the odds of the “unaware” group’s reported intentions/behaviors. -- The question was asked only in post-campaign survey.

**Table 5:** Support for the government’s efforts regarding obesity and sugary drinks among respondents in the pre-campaign and post-campaign periods, including a comparison of post-campaign respondents who were aware and unaware of the campaign.

	Pre-Campaign	Post-Campaign		Post-Campaign				
			*p*-Value	Unaware	Aware	*p*-Value	Adj. OR^ (95% CI)	*p*-Value
	(*n* = 1000)	(*n* = 1000)		(*n* = 453)	(*n* = 547)			
**Government Support (%)**								
It is important for my government to be involved in helping to solve the problem of obesity in South Africa	73	84	<0.001 *	84	83	0.694	0.92 (0.65, 1.30)	0.631
I intend to support government efforts to increase children’s access to healthy foods and drinks	80	84	0.014 *	85	84	0.534	0.89 (0.63, 1.27)	0.530
Government actions to reduce the public’s access to sugary drinks will hurt our economy	70	69	0.961	72	68	0.185	0.81 (0.62, 1.07)	0.144
The government should pass and enforce policies that discourage the consumption of junk food and sugary drinks	67	76	<0.001 *	78	75	0.244	0.89 (0.65, 1.20)	0.429
I support public education advertising campaigns that warn about the damages of sugary drinks and junk food on your health	79	81	0.344	78	83	0.023 *	1.50 (1.09, 2.08)	0.013 *
I support restrictions on the sale and/or provision of sugary drinks and junk food in school	66	74	<0.001 *	70	77	0.015 *	1.48 (1.11, 1.98)	0.009 *
I support banning or restricting advertising and/or marketing of sugary drinks and junk foods that is targeted at children	63	69	0.005 *	65	72	0.008 *	1.56 (1.18, 2.06)	0.002
I support requiring clear labels on the front of food and beverage packages that tell consumers if products are high in sugar, salt or fat	80	81	0.339	80	82	0.474	1.17 (0.84, 1.62)	0.355
I support a ban on the marketing or advertising of junk food/sugary drinks on school property and at school activities	61	69	<0.001 *	65	73	0.007 *	1.54 (1.17, 2.03)	0.002 *
I support a tax on sugary drinks if the money collected were invested in public programmes	62	70	<0.001 *	65	74	0.002 *	1.67 (1.26, 2.20)	<0.001 *
As part of its plan to address obesity in South Africa, the Department of Health recommends increasing the tax on sugary drinks, and the Department of Finance/Treasury has proposed a tax on sugar content that amounts to a roughly 20% tax on sugary drinks. Do you support or oppose the government’s proposal to tax sugary drinks?	43	58	<0.001 *	54	62	0.012 *	1.46 (1.13, 1.89)	0.004 *

* Significant difference at *p* < 0.05. Abbreviations: OR, Odds Ratio; CI, Confidence Interval. ^ Covariates adjusted for include age, gender, living standard measure, frequency of watching TV. A significant adjusted OR indicates that even after confounding factors have been taken into account, the odds of the “aware” group’s reported intentions/behaviors are significantly different from the odds of the “unaware” group’s reported intentions/behaviors.

**Table 6:** Behaviors and behavioral intentions among respondents in the pre-campaign and post-campaign periods, including a comparison of post-campaign respondents who were aware and unaware of the campaign.

	Pre-Campaign	Post-Campaign	*p*-Value	Post-Campaign				
				Unaware	Aware	*p*-Value	Adj. OR^(95% CI)	*p*-Value
	(*n* = 1000)	(*n* = 1000)		(*n* = 453)	(*n* = 547)			
**Behaviors and Behavioral Intentions**								
Thought of the health-related harms of being overweight/obese? (% often)	45	48	0.116	46	49	0.344	1.15 (0.89, 1.49)	0.288
Thought of the harms to your health of consuming sugary drinks? (% often)	44	50	0.003 *	52	49	0.419	0.95 (0.73, 1.23)	0.691
I would like my family to reduce their consumption of sugary drinks (% agree)	67	79	<0.001 *	78	81	0.257	1.35 (0.98, 1.86)	0.064
I intend to… (% agree)								
Reduce my consumption of sugary drinks	66	74	<0.001 *	73	75	0.490	1.14 (0.85, 1.54)	0.378
Reduce how often I offer sugary drinks to a child	65	74	<0.001 *	72	75	0.362	1.25 (0.93, 1.68)	0.133
% confident in ability to…			<0.001 *					
Reduce my consumption of sugary drinks	65	73	<0.001 *	73	73	0.994	1.03 (0.77, 1.38)	0.830
Reduce my child’s consumption of sugary drinks	66	78	<0.001 *	76	79	0.341	1.30 (0.85, 1.97)	0.225
% likely to...								
Reduce my consumption of sugary drinks in the next seven days	52	58	0.003 *	60	57	0.331	0.95 (0.73, 1.23)	0.681
Reduce my child’s consumption of sugary drinks in the next seven days	54	64	<0.001 *	62	66	0.392	1.32 (0.91, 1.91)	0.143
Reduction in consumption of sugary drinks compared to six months ago	23	27	0.020 *	27	27	0.809	0.98 (0.74, 1.31)	0.913
Increase in consumption of water compared to six months ago	--	42	--	39	45	0.048 *	1.34 (1.03, 1.74)	0.027 *

* Significant difference at *p* < 0.05. Abbreviations: OR, Odds Ratio; CI, Confidence Interval. ^ Covariates adjusted for include age, gender, living standard measure, frequency of watching TV. A significant adjusted OR indicates that even after confounding factors have been taken into account, the odds of the “aware” group’s reported intentions/behaviors are significantly different from the odds of the “unaware” group’s reported intentions/behaviors. -- The question was asked only in post-campaign survey.
